# Peritumoral Adipose Tissue Density Predicts the Malignancy in cT1 Renal Masses

**DOI:** 10.5152/tud.2023.23016

**Published:** 2023-05-01

**Authors:** Yusuf Sahin, Sinan Aygan, Ibrahim Hacibey, Murat Yuce, Mehmet Yilmaz, Sule Ozsoy, Atilla Semercioz, Ahmet Yaser Muslumanoglu

**Affiliations:** 1 Department of Urology, University of Health Sciences, Bağcılar Training and Research Hospital, Istanbul, Turkey; 2 Department of Radiology, University of Health Sciences, Bakırköy Dr. Sadi Konuk Training and Research Hospital, Istanbul, Turkey; 3 Department of Radiology, University of Health Sciences, Bağcılar Training and Research Hospital, Istanbul, Turkey; 4 Department of Pathology, University of Health Sciences, Bağcılar Training and Research Hospital, Istanbul, Turkey

**Keywords:** Adipose tissue, nephrectomy, renal cell cancer, tumor microenvironment

## Abstract

**Objective:**

Not only the frequency of surgery for small renal masses has increased secondary to the improvements and frequent use of imaging techniques but also the frequency of detection of benign lesions in nephrectomy specimens has increased as well. We aimed to assess the predictive value of computed tomography density of perirenal adipose tissue and peritumoral adipose tissue in distinguishing between benign and malignant renal masses.

**Materials and Methods:**

The current study included 116 patients who underwent nephrectomy for renal masses between January 2015 and December 2020. Clinicodemographic and preoperative computed tomography features and final pathological findings of the patients were recorded. According to the final pathological results, the patients were divided into 2 groups benign (n = 32) and malignant (n = 84). Groups were compared statistically in terms of perirenal adipose tissue and peritumoral adipose tissue density.

**Results:**

The median tumor size was 5.00 cm. The rate of benign tumors was higher in female patients (*P* = .005). The median peritumoral adipose tissue density among cT1 and cT1a tumors was higher in the malignant group (*P* < .001, for each). At a cutoff value of 97.50 Hounsfield Units, the peritumoral adipose tissue density had a sensitivity of 83.0% and a specificity of 79.2% for predicting the presence of malignant tumors in ≤7 cm renal masses. Using a cutoff value of −97.50 Hounsfield Units, the peritumoral adipose tissue density had a sensitivity of 88.9% and a specificity of 83.3% for predicting the presence of malignant tumors in ≤4 cm renal masses.

**Conclusion:**

The peritumoral adipose tissue density in the preoperative computed tomography images predicts the malignancy in cT1 renal masses.

**Main Points**

- With frequent usage of imaging modalities, the number of surgical interventions for small renal masses is increasing.
- As a result, the frequency of detection of benign lesions in pathological specimens is increasing.
- The present study demonstrated that peritumoral adipose tissue density on preoperative computed tomography images was significantly higher in the malignant renal masses in patients undergoing surgery for cT1 kidney tumor.
- We think that our study results may contribute to reduce surgical interventions for benign small renal masses by using peritumoral adipose tissue density on preoperative computed tomography images.

## Introduction

Frequently used imaging modalities have led to increased rates of surgical intervention, such as partial/radical nephrectomy, especially for small renal masses (SRMs).^1^ Renal masses which are suspected to be malignant on computed tomography (CT) or magnetic resonance imaging (MRI) are often surgically removed without a prior histological diagnosis. The additional value of renal mass biopsy (RMB), which is helpful to determine the pathological diagnosis, is still controversial. The non-diagnostic rate and low negative predictive value of the biopsy are the main concerns.^2^

Several studies have found a positive correlation between tumor size and the probability of malignancy.^3,4^ The rate of histologically confirmed benign tumors was 20% and 6% for ≤4 cm and >7 cm in tumor diameters, respectively.^3^ Unnecessary surgeries for benign lesions may increase the risk of psychosocial distress and operative morbidity of the patients.^5^ Although there are several studies investigating the clinical features, serum parameters, and radiological characteristics to predict the benign pathology in the final nephrectomy specimens, the results are still insufficient.^6-8^ Computed tomography remains the most effective cross-sectional imaging modality for the determination of renal tumor characteristics. However, even the enhancement pattern of the mass, which is the most promising CT feature, is insufficient with differentiation between benign and malignant renal masses. Although there are various CT characteristics such as central scar for oncocytoma and washout kinetics for angiomyolipoma (AML), they are not specific for them and may not always be present in these benign tumors.^8,9^

The adipose tissue (AT) mostly consists of adipocytes and was considered as an energy storage organ. However, it also consists of several cell types such as immunocytes, endothelial cells, and fibroblasts and secretes hormones, growth factors, chemokines, and adipokines, such as an endocrine organ.^10,11^ The AT may induce inflammation in the peritumoral microenvironment and provide energy to tumor cells with various secretions and plays an important role in the progression of many cancers.^12-14^ The CT density of AT, which is measured on non-contrast CT images, is one of the most widely used imaging features indicating the inflammatory and fibrotic changes in AT. It has been shown that CT density of the perirenal adipose tissue (PRAT) may predict the invasion into perinephric fatty tissue in patients with renal cell cancer (RCC) .^15^ In addition, increased peritumoral adipose tissue density (PTAT) on CT images has been shown to be inversely associated with recurrence-free survival in operated breast cancer patients.^16^

In this study, we hypothesized that increased PRAT and PTAT densities in preoperative CT images could predict the malignancy in final nephrectomy specimens. Therefore, we aimed to investigate predictive value of PRAT and PTAT densities in distinguishing between benign and malignant renal masses.

## Materials and Methods

### Study Design and Study Population

After obtaining Bağcılar Training and Research Hospital Clinical Research Ethics Committee approval (IRB No:2020.12.2.11.197.r1.211), the medical data of 245 patients who were performed radical/partial nephrectomy for renal masses at the department of urology of a tertiary referral hospital, were retrospectively reviewed between January 2015 and December 2020. The study was carried out in accordance with the Helsinki Protocol. Written informed consent was signed by the patients before surgery.

**Figure 1.**
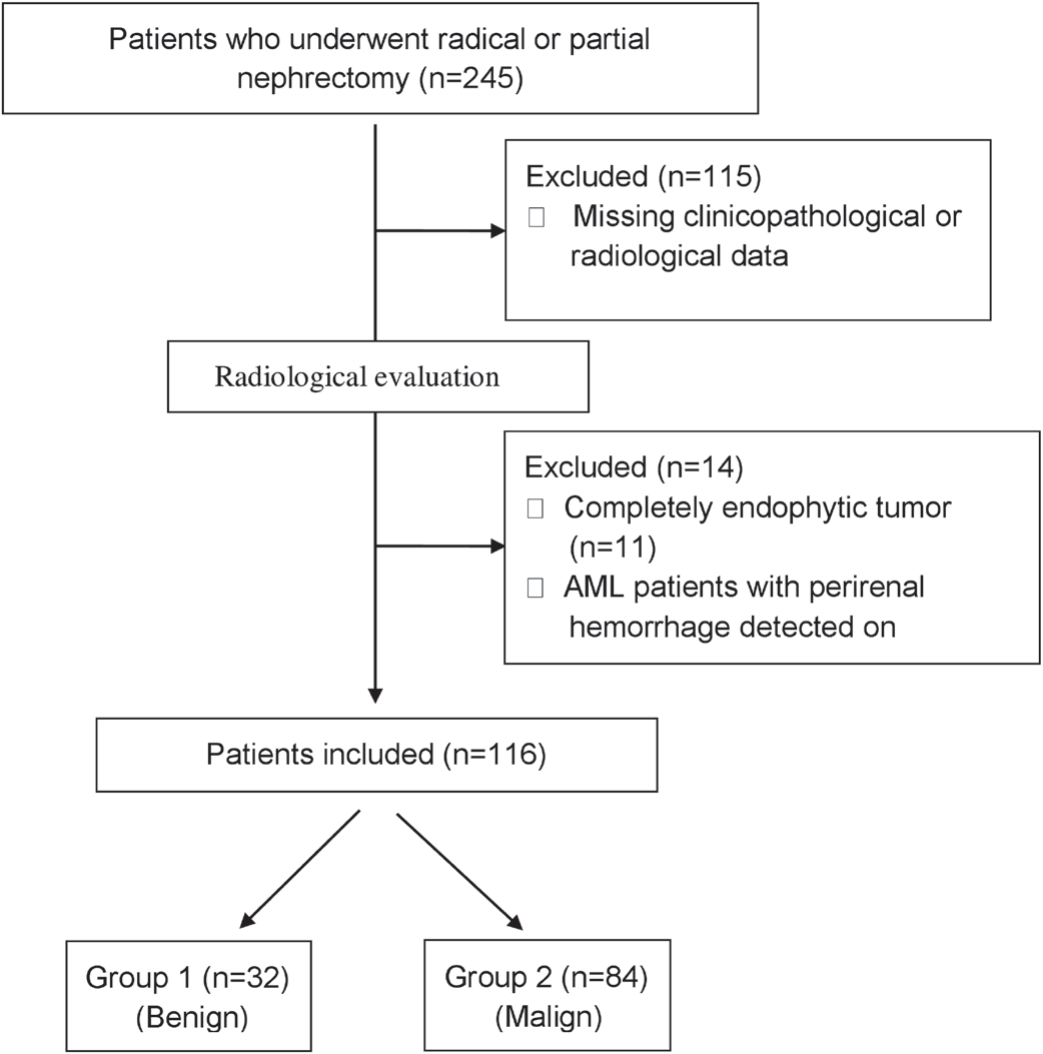
Flowchart of the study sample.

Preoperative clinicodemographical characteristics and biochemical results, radiological findings (tumor size, side, and location, PRAT densities and thicknesses for the tumoral and the contralateral non-tumoral kidney sides, PTAT density and thickness), surgery type, duration of hospital stay, and pathological features of nephrectomy specimens were recorded. Patients who had missing clinicopathological or radiological data (n = 115) and completely endophytic tumors whose PTAT density could not be measured (n = 11) were excluded. Three AML patients were also excluded, as perirenal hemorrhage was detected in the preoperative CT imaging. Finally, 116 patients were included. The patients were separated into 2 groups according to pathological features the benign (group 1, n = 32) and malignant (group 2, n = 84) groups. Flowchart of the study sample is shown in Figure 1.

### Computed Tomography Measurements

**Figure 2.**
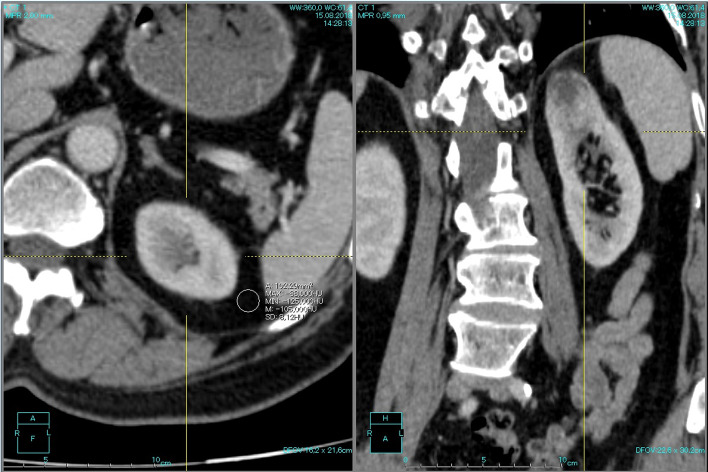
Perirenal adipose tissue density measurement in the upper pole.

**Figure 3.**
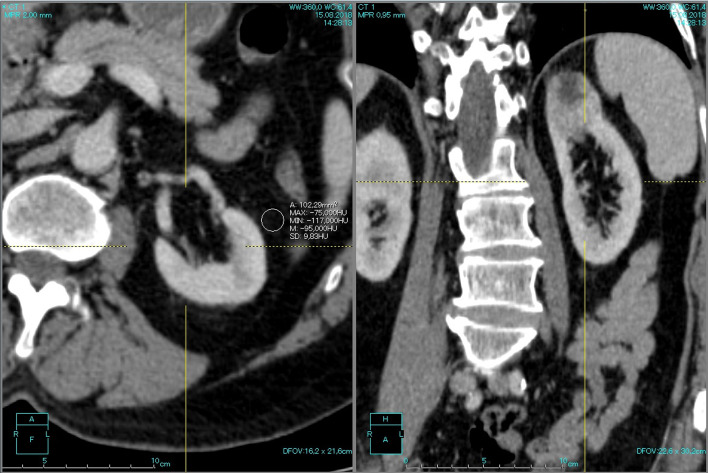
Perirenal adipose tissue density measurement in the middle zone.

**Figure 4.**
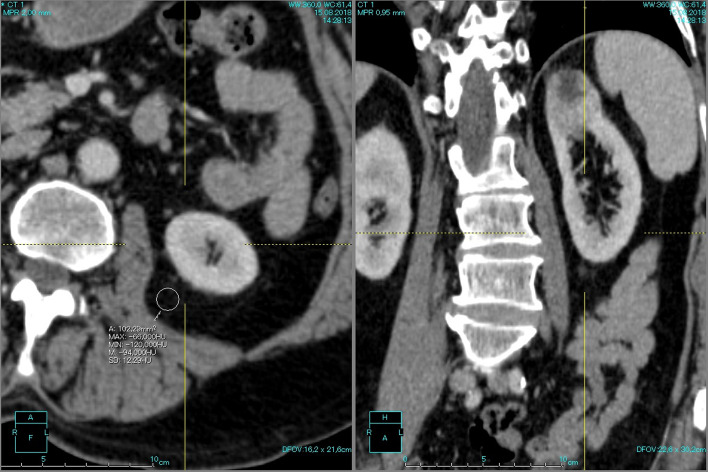
Perirenal adipose tissue density measurement in the lower pole.

**Figure 5.**
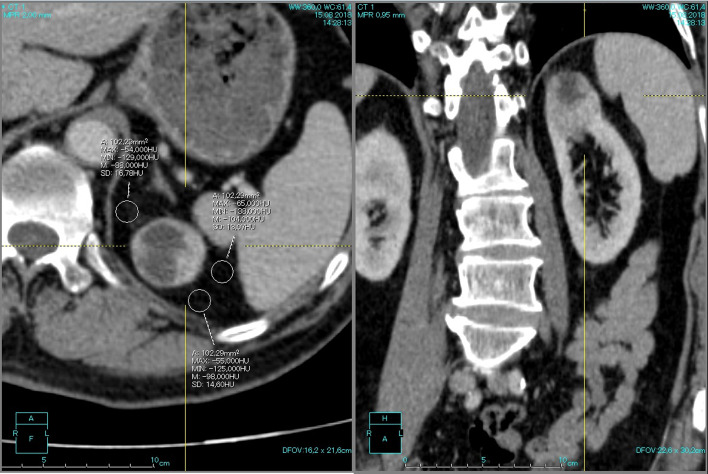
Peritumoral adipose tissue density measurement.

All CT examinations were performed in a supine position with 128 Ingenuity (Philips Healthcare, the Netherlands) device. The unenhanced images of preoperative abdominal dynamic CT were analyzed by 2 radiologists in agreement, who were blinded to the clinical data of the patients. Serial sections of 5 mm thickness were obtained from all patients, including the 10th thoracic vertebra and the symphysis pubis space. Perirenal fatty tissues were separated from other tissues according to their Hounsfield Units (HU) rate. The regions of interest (ROI) with a width of approximately 10 mm^2^ were measured from the PRAT in the lower, middle, and upper zones of both kidneys, and the average densities were recorded as the tumor side and the normal contralateral side PRAT density, respectively (Figures 2-4). In addition, the PTAT density was measured by taking the average of 3 separate ROIs with a width of approximately 10 mm^2^ at a distance of approximately 3 mm from the discernible tumor margin (Figure 5). The PRAT thickness was measured as the shortest distance from the outer border of the renal cortex to the inner border of the posterior abdominal wall. Measurements for the tumor side and the normal contralateral side kidney were made separately in the upper, middle, and lower zones in axial sections and the average of these values was recorded as the PRAT thickness for each side.

### Statistical Analysis

Statistical Package for the Social Sciences (SPSS) version 25.0 software (IBM SPSS Corp., Armonk, NY, USA) was used. The Kolmogorov-Smirnov and Shapiro-Wilk tests were used to evaluate the normality of data in quantitative variables. Median and interquartile range (IQR) were used for continuous variables, while number and frequency were used for categorical variables. The qualitative data of the groups were compared by the Pearson chi-square and Fisher exact tests. The radiological features of the groups, such as PRAT densities and thicknesses, were compared with the Mann–Whitney *U* test. The optimal cutoff value for tumor side PRAT and PTAT densities to predict the malignancy was determined by the receiver operating characteristic (ROC) curve analysis. The sensitivity, specificity, positive and negative predictive values were determined for the optimal cutoff values. A two-tailed *P*-value of <.05 was considered statistically significant.

## Results

There were 32 (27.6%) patients in group 1 and 84 (72.4%) patients in group 2. Demographic, preoperative clinical and tumor characteristics, and perioperative surgical parameters of the patients are summarized in Table 1. Angiomyolipoma was the most common subtype in group 1 with a rate of 43.8%. Final pathological specimen results of the patients are summarized in Table 2.

The median tumor side PRAT and PTAT densities among all patients were higher in group 2 (−98.16 HU vs. −102.50 HU; *P* = .002, and −87.50 HU vs. −100.50 HU; *P* < .001, respectively). Among cT1 tumors, the median PTAT density was higher in group 2 (−91.00 HU vs. −102.50 HU; *P* < .001). The median PTAT density was also higher in group 2 among cT1a tumors (−90.00 HU vs. −104.50 HU; *P* < .001) (Table 3).

**Figure 6.**
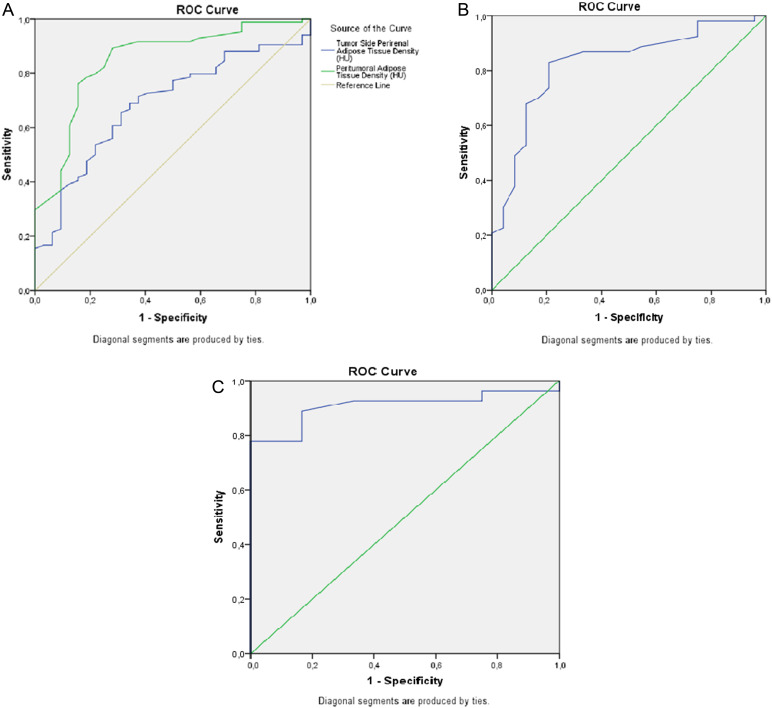
Receiver operating characteristic (ROC) curve analysis. (A) Receiver operating characteristic curve analysis for tumor side perirenal adipose tissue density and peritumoral adipose tissue (PTAT) density in all patients. (B) Receiver operating characteristic curve analysis for PTAT density in ≤7 cm renal masses. (C) Receiver operating characteristic curve analysis for PTAT density in ≤4 cm renal masses.

The ROC curve analysis showed that the cutoff values of tumor side PRAT and PTAT densities for the presence of malignant tumors in all patients were −101.15 HU and −96.00 HU, respectively. The area under curves (AUCs) were 0.687 (95% CICI = 0.584-0.789, *P* =.002) and 0.847 (95% CI = 0.766-0.928, *P* < .001) for PRAT and PTAT densities, respectively (Figure 6A). The tumor side PRAT density cutoff value (−101.15 HU) had a sensitivity of 69.0% and a specificity of 65.6% for predicting the presence of malignant tumors. The PTAT density cutoff value (−96.00 HU) had a sensitivity of 82.1% and a specificity of 75.0% for predicting the presence of malignant tumors. At a cutoff value of −97.50 HU, the PTAT density had a sensitivity of 83.0% and a specificity of 79.2% for predicting the presence of malignant tumors in ≤7 cm renal masses. The AUC level was 0.825 (95% CI = 0.724-0.926, *P* < .001) (Figure 6B). Using a cutoff value of −97.50 HU, the PTAT density had a sensitivity of 88.9% and a specificity of 83.3% for predicting the presence of malignant tumors in ≤4 cm renal masses. The AUC level was 0.907 (95% CI = 0.811-1.000, *P* < .001) (Figure 6C).

## Discussion

This study showed that the PRAT and PTAT densities were significantly higher in malignant renal masses. In addition, PTAT density was significantly higher in both cT1 and cT1a malignant renal masses. The same cutoff value (−97.50 HU) for PTAT has a statistically significant sensitivity and specificity for predicting both cT1 and cT1a malignant renal masses. Moreover, its predictive value for malignancy according to AUC was higher particularly in SRMs. With the common use of imaging modalities, the number of surgical interventions for SRMs is increasing. As a result, the frequency of detection of benign lesions in pathological specimens is increasing. We think that our study results may contribute to reduce surgical interventions for benign SRMs by using PTAT density on preoperative CT images.

The frequency of benign pathology in renal masses is 25%-32% in the literature.^8,17,18^ In the current study, the rate of benign pathology in all patients and SRMs was 27% and 30.8%, respectively, which is in concordance with the literature. This rate suggests that approximately 1 in 4 suspected renal masses are treated surgically rather than followed up. Due to the high rate of benign pathology in cT1 renal masses, investigation of the factors that may predict the malignancy has always been a matter of interest. In a study by Zisman et al.^19^ among 1664 patients who underwent nephrectomy for kidney tumor, female sex and young age in females were found to be predictive factors for benign histology, and the rate of benign tumor was 21% in females and 13% in males. Similarly, a recent multi-center study demonstrated that female sex, partial nephrectomy, and lower BMI were more frequent in benign pathology.^7^ In our study, the frequency of benign histology was higher in females than in males (2 : 1 ratio). However, unlike previous studies, BMI did not differ significantly between groups. We believe that the lack of statistically significant difference between the groups in terms of PRAT and PTAT thicknesses supports this finding. The relationship between obesity and RCC is well known, and one of the underlying causes of obesity is impaired lipid metabolism. Therefore, a number of studies have investigated the possible relationship between lipid levels and RCC. The Swedish Apolipoprotein Mortality Risk Study (AMORIS) showed a significant relationship between RCC and triglyceride levels.^20^ In a recent study, the plasma atherogenic index, which is the ratio of serum triglyceride to high-density lipoprotein cholesterol, was found to be significantly higher in malignant masses compared to benign ones in patients who underwent partial or radical nephrectomy for Bosniak 3 and 4 renal cysts.^21^ Recently, it has been shown that visceral adiposity reflects the lipid metabolism disorder with higher accuracy. Visceral AT is more active than subcutaneous AT in terms of metabolic and hormonal activity and plays a more active role in carcinogenesis.^22^ Based on the link between visceral obesity and RCC, there are several studies investigating the relationship between PRAT volume or thickness and RCC. Okhunov et al^23^ revealed that perirenal fat distance might predict the clear cell subtype of RCC in final pathology results of patients who underwent partial nephrectomy for SRMs. A recent study including 153 patients with cT1 RCC who underwent nephrectomy showed that the increased perirenal fat percentage could predict the protrusion of tumor to the perirenal area.^24^ In the present study, PRAT or PTAT thicknesses between benign and malignant tumor groups were not significantly different. Since there are many factors affecting the amount of visceral fat, the quantitative features of PRAT such as thickness or volume may not accurately reflect the tumor microenvironment.^25^ Therefore, visceral, subcutaneous, or PRAT densities as qualitative factors were investigated in predicting the invasion and prognosis of RCC and other cancers, recently. Lee et al^26^ demonstrated that higher pretreatment CT density of visceral AT and subcutaneous AT, and fluorodeoxyglucose uptake of visceral AT in positron emission tomography were associated with worse overall survival in pancreatic adenocarcinoma patients. Tsili et al^15^ also found that the PRAT and renal sinus densities on preoperative CT were higher in patients with perirenal invasion than in tumors confined to the kidney or in a healthy contralateral kidney in nephrectomy patients. However, in the regression analysis, nodular lesions in PRAT as evidenced by contrast-enhanced CT were found to be the only factors that could predict perirenal invasion. Perirenal invasion is very rare, particularly in small malignant tumors, and the peritumoral microenvironment may provide more accurate information about the presence of malignancy rather than invasion in these tumors. As the tumor size grows, the inflammation in the peritumoral area may be replaced by the invasion of tumor cells. However, there is a high probability that there would be no significant difference between the CT density of the area of inflammation and tumor cells. Therefore, PRAT density may not predict the invasion in malignant tumors, regardless of tumor size. In our study, regardless of tumor size, PRAT and PTAT densities were higher in the malignant tumors, while only PTAT density was found to be significantly higher in particularly malignant SRMs. Suspicious lesions with a PTAT density less than −97.5 HU may be candidates for preoperative RMB according to this study's results. Thus, unnecessary surgery may be prevented by recommending follow-up for tumors with benign histology according to the biopsy result.

Nonetheless, this study has some limitations and the most important of which is its retrospective design. Second, the missing data rate is a bit high considering the study population. The main reason for this situation was being a tertiary referral center. The radiological images of some patients who were referred to our clinic from an external center were not recorded in our hospital data system. Third, PRAT and PTAT densities were not compared between RCC subtypes as the small number of papillary and chromophobe RCC patients. On the other hand, this is the first study that investigated the predictive value of PRAT and PTAT densities for malignancy in renal masses.

In conclusion, the present study demonstrated that PTAT density on preoperative CT images was significantly higher in the malignant renal masses in patients undergoing surgery for cT1 kidney tumors. We think that our study results may contribute to reduce surgical interventions for benign SRMs by using PTAT density on preoperative CT images.

## Figures and Tables

**Table 1.** Demographic, Preoperative Clinical and Tumor Characteristics and Perioperative Surgical Parameters

Variable(s)		All Patients (n = 116, 100%)	Group 1 (Benign) (n = 32, 27.6%)	Group 2 (Malignant) (n = 84, 72.4%)	*P* ^Ψ^
		n (%)	n (%)	n, %	
Age (year) (median [IQR])		57 (48-66)	56 (51-63)	57 (47-67)	.512^ a^
Sex	Female	52 (44.8%)	21 (65.6%)	31 (36.9%)	.005^*, b^
	Male	64 (55.2%)	11 (34.4%)	53 (63.1%)	
BMI (kg/m2) (median [IQR])		30.0 (25.0-34.0)	31.5 (26.5-35.0)	30.0 (25.0-34.0)	.469^ a^
Hypertension (yes)		46 (39.7%)	12 (37.5%)	34 (40.5%)	.770^ b^
Diabetes mellitus (yes)		26 (22.4%)	3 (9.4%)	23 (27.4%)	.038^*,b^
Coronary artery disease (yes)		17 (14.7%)	3 (9.4%)	14 (16.7%)	.393^ c^
Cerebrovascular accident (yes)		3 (2.6%)	1 (3.1%)	2 (2.4%)	1.000^ c^
ASA class	ASA class-1	72 (62.1%)	11 (34.4%)	61 (72.6%)	<.001^*, c^
	ASA class-2	36 (31.0%)	16 (50.0%)	20 (23.8%)	
	ASA class-3	7 (6.0%)	5 (15.6%)	2 (2.4%)	
	ASA class-4	1 (0.9%)	0	1 (1.2%)	
Preoperative creatinine (mg/dL) (median [IQR])		0.80 (0.69-1.00)	0.81 (0.70-1.01)	0.79 (0.67-0.97)	.306^ a^
Tumor side	Right	53 (45.7%)	15 (46.9%)	38 (45.2%)	.874^ b^
	Left	63 (54.3%)	17 (53.1%)	46 (54.8%)	
Tumor localization	Lower pole	40 (34.5%)	13 (40.6%)	27 (32.1%)	.355^b^
	Middle pole	39 (33.6%)	12 (37.5%)	27 (32.1%)	
	Upper pole	37 (31.9%)	7 (21.9%)	30 (35.7%)	
Tumor diameter (cm) (median [IQR])		5.00 (3.50-8.00)	5.00 (3.00-7.50)	5.00 (3.50-8.00)	.460^ a^
Surgery type	Nephron-sparing surgery	47 (40.5%)	19 (59.4%)	28 (33.3%)	.011^*, b^
	Radical nephrectomy	69 (59.5%)	13 (40.6%)	56 (66.7%)	
Surgical approach	Open	63 (54.3%)	10 (31.3%)	53 (63.1%)	.002^*,b^
	Laparoscopic	53 (45.7%)	22 (68.8%)	31 (36.9%)	
Length of hospital stay (day) (median [IQR])		3 (1-4)	5 (3-6)	2 (1-4)	<.001^*,a^

^Ψ^*P* values describe the comparison of Benign and Malignant groups.

^a^Mann–Whitney *U* test;^ b^Pearson Chi-square test; ^c^Fisher’s exact test; ^*^P < .05.

ASA, American Society of Anesthesiologists; BMI, Body Mass Index; IQR, interquartile range.

**Table 2.** Final Pathological Specimen Results of the Patients

Variable(s)			n (%)
Benign/Malignant	Benign		32 (27.6)
	Malignant		84 (72.4)
Pathology	Benign cystic lesion		11 (9.5)
	Angiomyolipoma		14 (12.1)
	Oncocytoma		7 (6.0)
	Renal cell carcinoma		82 (70.7)
		Clear cell	61 (52.6)
		Papillary	16 (13.8)
		Chromophobe	5 (4.3)
	Neuroendocrine tumor		1 (0.9)
	Primitive neuroectodermal tumor		1 (0.9)
Pathological tumor stage	pT1a		27 (23.3)
	pT1b		26 (22.4)
	pT2a		9 (7.8)
	pT2b		4 (3.4)
	pT3a		17 (14.7)
	pT3b		0
	pT4a		1 (0.9)
Fuhrman grade	Grade-1		4 (3.4)
	Grade-2		36 (31.0)
	Grade-3		28 (24.1)
	Grade-4		9 (7.8)
Surgical margin	Negative		102 (87.9)
	Positive		14 (12.1)
Sarcomatoid differentiation (yes)			10 (8.6)
Necrosis (yes)			23 (19.8)
Lymphovascular invasion (yes)			22 (19.0)
Perineural invasion (yes)			2 (1.7)

**Table 3.** Comparison of All, ≤7 cm, and ≤4 cm Renal Masses in Terms of Radiological Measurement Parameters

Variable (s)	Group 1 (Benign) (n = 32, 27.6%)	Group 2 (Malignant) (n = 84, 72.4%)		Benign (n = 24, 31.2%)	pT1 (n = 53, 68.8%)		Benign (n = 12, 30.8%)	pT1a (n = 27, 69.2%)	
	Median (IQR)	Median (IQR)	*P* ^Ψ^	Median (IQR)	Median (IQR)	*P* ^Ψ^	Median (IQR)	Median (IQR)	*P* ^Ψ^
Tumor side PRAT density (HU)	−102.50 (−105.80 to −99.00)	−98.16 (−102.50 to −93.67)	.002^*,a^	−102.50 (−105.80 to −99.65)	−100.33 (−104.00 to −97.67)	.183^a^	−103.20 (−105.80 to −99.65)	−102.67 (−105.00 to −99.33)	.891^a^
Normal contralateral side PRAT density (HU)	−102.45 (−107.49 to −98.63)	−101.00 (−104.50 to −97.00)	.252^a^	−102.95 (−107.49 to −99.15)	−101.67 (−105.33 to −99.33)	.692^a^	−101.35 (−106.80 to −99.30)	−104.33 (−106.33 to −101.00)	.247^a^
Tumor side PRAT thickness (mm)	13.30 (8.97 to 16.67)	11.90 (9.13 to 17.84)	.853^a^	13.30 (8.97 to 17.90)	11.72 (9.11 to 17.60)	.660^a^	14.66 (11.52 to19.77)	13.78 (9.25 to18.26)	.447^a^
Normal contralateral side PRAT thickness (mm)	12.60 (9.07 to 15.65)	11.55 (8.81 to 14.55)	.503^a^	13.60 (9.49 to 15.85)	11.94 (8.99 to 15.85)	.325^a^	14.93 (12.70 to 16.88)	13.15 (8.99 to 16.20)	.140^a^
PTAT density (HU)	−100.50 (−107.00 to −96.00)	−87.50 (−92.00 to −79.00)	<.001^*,a^	−102.50 (−107.00 to −98.00)	−91.00 (−97.00 to −86.00)	<.001^*,a^	−104.50 (−107.50 to −98.00)	−90.00 (−93.00 to −86.00)	<.001^*,a^

^Ψ^*P* values describe the comparison of benign and malignant groups.

^a^Mann–Whitney *U* test, ^*^P < .05.

HU, Hounsfield units; IQR, interquartile range; PRAT, perirenal adipose tissue; PTAT, peritumoral adipose tissue.
